# One-year Mediterranean diet promotes epigenetic rejuvenation with country- and sex-specific effects: a pilot study from the NU-AGE project

**DOI:** 10.1007/s11357-019-00149-0

**Published:** 2020-01-24

**Authors:** Noémie Gensous, Paolo Garagnani, Aurelia Santoro, Cristina Giuliani, Rita Ostan, Cristina Fabbri, Maddalena Milazzo, Davide Gentilini, Anna Maria di Blasio, Barbara Pietruszka, Dawid Madej, Agata Bialecka-Debek, Anna Brzozowska, Claudio Franceschi, Maria Giulia Bacalini

**Affiliations:** 1 Department of Experimental, Diagnostic and Specialty Medicine (DIMES), Alma Mater Studiorum – University of Bologna, Via San Giacomo 12, 40126 Bologna, Italy; 2 Center for Applied Biomedical Research (CRBA), St. Orsola-Malpighi University Hospital, Bologna, Italy; 3 Clinical Chemistry, Department of Laboratory Medicine, Karolinska Institutet at Huddinge University Hospital, S-141 86 Stockholm, Sweden; 4 CNR Institute of Molecular Genetics “Luigi Luca Cavalli-Sforza”, Unit of Bologna, Bologna, Italy; 5 Department of Biological, Geological, and Environmental Sciences (BiGeA), Laboratory of Molecular Anthropology and Centre for Genome Biology, University of Bologna, Bologna, Italy; 6 Department of Brain and Behavioral Sciences, University of Pavia, Pavia, Italy; 7 Istituto Auxologico Italiano IRCCS, Cusano Milanino, Milan, Italy; 8 Department of Human Nutrition, Warsaw University of Life Sciences-SGGW, Warsaw, Poland; 9 Laboratory of Systems Medicine of Healthy Aging and Department of Applied Mathematics, Lobachevsky Univeristy, Nizhny Novgorod, Russia; 10 IRCCS Istituto delle Scienze Neurologiche di Bologna, Bologna, Italy

**Keywords:** Epigenetics, DNA methylation, Epigenetic clock, Epigenetic age acceleration, Biological age, Mediterranean-like diet

## Abstract

Mediterranean diet has been proposed to promote healthy aging, but its effects on aging biomarkers have been poorly investigated. We evaluated the impact of a 1-year Mediterranean-like diet in a pilot study including 120 elderly healthy subjects from the NU-AGE study (60 Italians, 60 Poles) by measuring the changes in their epigenetic age, assessed by Horvath’s clock. We observed a trend towards epigenetic rejuvenation of participants after nutritional intervention. The effect was statistically significant in the group of Polish females and in subjects who were epigenetically older at baseline. A genome-wide association study of epigenetic age changes after the intervention did not return significant (adjusted *p* value < 0.05) loci. However, we identified small-effect alleles (nominal *p* value < 10–4), mapping in genes enriched in pathways related to energy metabolism, regulation of cell cycle, and of immune functions. Together, these findings suggest that Mediterranean diet can promote epigenetic rejuvenation but with country-, sex-, and individual-specific effects, thus highlighting the need for a personalized approach to nutritional interventions.

**Electronic supplementary material:**

The online version of this article (10.1007/s11357-019-00149-0) contains supplementary material, which is available to authorized users.

## Introduction

As the population continues to age within Europe, an increase in the incidence of age-related diseases is observed (WHO 2017). Indeed, the increasing lifespan is not associated with an increase in health span, and this issue represents a great challenge for our societies. There is an important need to identify factors that are able to influence health in old age and to develop and validate interventions that could slow down or counteract the process of aging and its associated pathologies. A possible strategy to impact on aging is to intervene on lifestyle factors, such as diet or physical activity. Nutritional interventions seem to be one of the most promising approaches to promote healthy aging, and growing amount of data indicates that they can influence the health status of subjects (Longo et al. 2015; Dato et al. 2016; Wahl et al. 2016; Shlisky et al. 2017; Xia et al. 2017; Heiss et al. 2017).

Mediterranean diet, which is considered by UNESCO as a heritage of humanity, is a well-balanced mix of nutrients, anti-oxidants, and anti-inflammatory molecules, and it has been recently suggested that some of its components are able to exert hormetic effects (Martucci et al. 2017). This diet has demonstrated favorable effects on cardiovascular risk, blood pressure, cancer, inflammation, or frailty status (Estruch et al. 2006, 2013; Mitjavila et al. 2013; Ostan et al. 2015; Martínez-González et al. 2015; Kojima et al. 2018), and it has been observed that it can impact methylation of inflammation-related genes in peripheral blood cells (Arpón et al. 2017). Some studies have suggested that Mediterranean diet prevents telomere shortening, a well-established biomarker of age, but results are not consistent among different studies (Davinelli et al. 2019). The role of Mediterranean diet in promoting healthy aging has been recently investigated in the framework of the European project NU-AGE (“New dietary strategies addressing the specific needs of elderly population for an healthy aging in Europe” (http://www.nu-age.eu/)), a large multidisciplinary consortium with 30 partners across Europe (Berendsen et al. 2014; Santoro et al. 2014). The aim of NU-AGE project was to investigate how an intervention based on Mediterranean diet, specifically tailored according to the nutritional needs of people over 65 years of age, can impact on age-related diseases and functional decline, possibly modulating inflammaging and its outcomes (Franceschi et al. 2000). Probands were enrolled in five European countries (Italy, Poland, France, the Netherlands, and the UK), and a 1-year Mediterranean-like diet was administered to the intervention subgroup. A comprehensive clinical and molecular characterization was performed at baseline and after the 1-year intervention, and results achieved so far in the framework of this study have demonstrated a beneficial effect of the Mediterranean-like diet on global cognition and episodic memory (Marseglia et al. 2018), osteoporosis (Jennings et al. 2018), immune function (Maijo et al. 2018), and cardiovascular health (Jennings et al. 2019), as well as on the proteasomal proteolysis (Athanasopoulou et al. 2018). The NU-AGE study design (different countries with different dietary traditions and habits) and the large number of collected data allowed to evaluate the impact of relevant variables usually poorly investigated (age, sex, and ethnicity/genetics, as well as individual characteristics) on different parameters at baseline and after the intervention (Konz et al. 2018; Marseglia et al. 2018; Ostan et al. 2018; Pujos-Guillot et al. 2018; Santoro et al. 2018, 2019; Jennings et al. 2019). Importantly, the enrolled subjects were recruited also in non-Mediterranean countries (Poland, the Netherlands, and the UK). The effects of a Mediterranean diet intervention on non-Mediterranean countries is not granted, because its transferability requires specific changes in dietary habits (Martínez-González et al. 2017) and because genetic and environmental factors, that can be country-specific, can hamper/enhance its effects (Mayr et al. 2018).

In order to monitor the impact of anti-aging interventions, accurate biological measures of age are needed. The discovery of the so-called epigenetic clocks, based on DNA methylation (DNAm) levels at some specific sites, has been a major breakthrough in this field within the last 6 years (Horvath 2013; Hannum et al. 2013; Weidner et al. 2014). These biomarkers have been proposed as accurate and robust biomarkers of aging, but also as indicators of the biological health of an individual: DNAm age, also known as epigenetic age, measured in blood cells, has been found to be predictive of mortality (Marioni et al. 2015a; Perna et al. 2016; Christiansen et al. 2016; Chen et al. 2016; Dugué et al. 2018) and other aging-related outcomes such as frailty (Breitling et al. 2016) or cognitive and physical functioning (Marioni et al. 2015b; Degerman et al. 2017; Simpkin et al. 2017; Gale et al. 2018a; Sillanpää et al. 2018). Recent studies demonstrated that age-associated epigenetic variations can be affected by diet (Bacalini et al. 2014; Kok et al. 2015; Quach et al. 2017) and that exposure to some pathogenic conditions or environmental factors can influence DNAm age (Horvath et al. 2014; Nevalainen et al. 2017; Li et al. 2018; Rosen et al. 2018); however, little is known about the specific relationship between nutritional interventions and epigenetic biomarkers of aging.

In the present manuscript, we used epigenetic biomarkers of aging to study the impact of the nutritional intervention delivered in the framework of NU-AGE project, focusing on subjects enrolled in a Mediterranean country (Italy) and in a non-Mediterranean country (Poland).

## Subjects and methods

### NU-AGE study

NU-AGE was a 1-year, multicenter, randomized, single-blind, controlled trial (registered with *clinicaltrials.gov*, NCT01754012) with two parallel groups (i.e., dietary intervention and control) carried out during April 2012–January 2015 in five European centers in Italy, Poland, France, the Netherlands, and the UK. The recruitment of participants has been described in detail previously (Berendsen et al. 2014; Santoro et al. 2014). Briefly, 1279 participants aged 65–79 years, free of major overt chronic diseases for at least 2 years (i.e., cancer, severe organ disease), living independently, and free of dementia, were recruited to participate in the baseline assessment. At enrollment, exclusion criteria included severe heart diseases, type 1 and insulin-treated type 2 diabetes, chronic use of corticosteroids, recent use of antibiotics, change in habitual medication use, frailty (Fried et al. 2001), malnutrition (body mass index (BMI) < 18.5 kg/m^2^ or 10% weight loss within 6 months), or food allergy/intolerance requiring special diets. Participants were randomly assigned (1:1) to the control or intervention groups, after stratification by sex, age (65–72 or > 72–79 years), frailty status (pre-frail or non-frail), and body mass index (< 25 or ≥ 25 kg/m^2^). All participants provided written informed consent.

Epigenetic analysis was performed in a subgroup of 120 randomly selected subjects (60 from the Italian cohort and 60 from the Polish one) from the intervention group, at both baseline (T0) and after 1 year of dietary intervention (T1), for a total of 240 samples. Exact chronological age of the subjects (in years) at T0 was calculated as follows: [(T0 date) − (date of birth)]/365. Exact chronological age at T1 was calculated as follows: [(T1 date) − (date of birth)]/365. Adherence to study protocol was evaluated using the 7-day food records. A NU-AGE index scoring system was specifically developed and used as a measure of adherence to the NU-AGE diet, as described previously (Jennings et al. 2018).

### Analysis of DNA methylation

Samples were analyzed for genome-wide DNA methylation patterns using the Illumina Infinium HumanMethylation450 BeadChip array (Illumina Inc., CA, USA). Genomic DNA was extracted from 250 μL of whole blood (drawn on EDTA tubes), using the QIAamp 96 DNA Blood Kit (QIAGEN, Hilden, Germany). One microgram of DNA was bisulfite converted, using the EZ DNA Methylation Kit (Zymo Research Corporation, Orange, CA, USA) according to manufacturer’s instructions. After bisulfite conversion, DNA was whole-genome amplified, enzymatically fragmented, and hybridized to the Illumina Infinium HumanMethylation450 BeadChips (Illumina Inc., CA, USA), according to the manufacturer’s protocols. Samples from the different groups (Italy and Poland, T0 and T1) were accurately randomized across the experimental sessions. Arrays were scanned using the HiScan instrument (Illumina Inc., CA, USA). Raw fluorescence intensities were extracted using *minfi* Bioconductor package, and normalization was performed using the *preprocessQuantile* function (Touleimat and Tost 2012).

### Evaluation of DNA methylation age and of epigenetic age acceleration

Normalized DNA methylation data were uploaded into the DNA methylation age calculator, freely available at the website: https://dnamage.genetics.ucla.edu, to calculate DNAm age, as described by Horvath (Horvath 2013). DNAm age is calculated using the weighted average of DNA methylation levels at 353 CpG sites (Horvath 2013). The “advanced blood analysis” option was selected in the online calculator, allowing the calculation of three measures of epigenetic age acceleration (AA) that were further considered in this study. These measures have been previously described by Horvath and colleagues and have been applied to date in several publications (Levine et al. 2015; Horvath and Ritz 2015; Horvath et al. 2015, 2016; Chen et al. 2016; Ambatipudi et al. 2017; Quach et al. 2017; Maierhofer et al. 2017; Gale et al. 2018a, 2018b). The first measure is considered as the universal measure of epigenetic AA and is denoted AgeAccel. It corresponds to the residual that results from regressing DNAm age on chronological age. The second measure of epigenetic AA is referred as Intrinsic Epigenetic Age Acceleration (IEAA), denoted as AAHOAdjCellCounts in the online software. IEAA is defined as the residual resulting from regressing DNAm age on chronological age and seven measures of immune blood cell count estimates: naive CD8+ T cells, exhausted CD8+ T cells, plasma B cells, CD4+ T cells, natural killer cells, monocytes, and granulocytes. IEAA is independent of changes in blood cell composition that occur with time and is considered as a measure of “pure” epigenetic aging effects in blood cells. Finally, the third measure considered is referred as Extrinsic Epigenetic Age Acceleration (EEAA), known as BioAge4HAStaticAdjAge in the online software. EEAA is based on a weighted average of the epigenetic age measure with Hannum’s clock (Hannum et al. 2013) and three blood cell types that are known to change with age: naive cytotoxic T lymphocytes (CD45RA+CCR7+), exhausted cytotoxic T lymphocytes (CD45RA-CD28-), and plasma B cells. EEAA is defined as the residual formed from regressing the resulting weighted epigenetic age on chronological age. This measure is dependent on age-related changes in blood cell composition and can be considered as a measure of aging in immune system.

### Estimating blood cell counts based on DNA methylation levels

Blood cell counts used in the measures of IEAA and EEAA were estimated based on DNA methylation data using the epigenetic clock online software. Blood cell proportions of CD8+ T cells, CD4+ T cells, natural killer cells, B cells, and granulocytes are based on Houseman’s estimation method (Houseman et al. 2012). An advanced analysis option of the epigenetic clock software is used to estimate the percentage of naïve and exhausted CD8+ T cells.

### Genotyping

Genomic DNA was extracted from 250 μL of whole blood (drawn on EDTA tubes), using the QIAamp 96 DNA Blood Kit (QIAGEN, Hilden, Germany). Two hundred nanograms of genomic DNA were genotyped for 713,014 genetic markers by the Illumina OmniExpress BeadChip (Illumina Inc., CA, USA), according to manufacturer’s protocol. After quality control, 118 samples were retained. Quantitative trait association analysis and estimation of single nucleotide polymorphisms’ (SNPs) allele frequencies were performed using PLINK toolset.

### Statistical analysis

The effects of the nutritional intervention on the three abovementioned measures of epigenetic AA (AgeAccel, IEAA, and EEAA) were analyzed with a Student’s paired-sample *t* test. For each epigenetic AA measure, Benjamini-Hochberg procedure was applied to correct for multiple tests, considering a total of 6 tests. Pearson correlations between measures of epigenetic age and chronological age or scores of adherence to Mediterranean diet were calculated. All statistical analyses and graphics were produced using the R v3.3.2.

## Results

### Subjects

Genome-wide DNA methylation profiles were analyzed by the Illumina Infinium HumanMethylation450 Beadchip (Illumina Inc., CA, USA) in whole blood of 120 European subjects belonging to the intervention group of the NU-AGE study, with chronological age ranging from 65 to 79 years old. Sixty patients were recruited in Italy and the other half was recruited in Poland. Characteristics of enrolled subjects are summarized in Table 1. Baseline characteristics were similar between the two groups in terms of chronological age and adherence to Mediterranean diet (Student’s *t* test *p* value > 0.05) (Supplemental Fig. 1). Body mass index (BMI) tended to be higher in Polish subjects compared with Italian ones, and this difference was statistically significant when considering only males (Student’s *t* test *p* value = 0.027) (Supplemental Fig. 1). After 1 year of nutritional intervention (T1), adherence to Mediterranean diet significantly increased in both Italian and Polish participants, and a significant decrease in BMI was observed in Italian males (paired Student’s *t* test *p* value = 0.008) (Supplemental Figs. 1 and 2).

**Table 1:** Characteristics of the study population at baseline (T0) and after 1 year of Mediterranean-like diet (T1)

Country			Italy		Poland	
Subjects (*n*)			60		60	
Males/females (*n*)			27/33		24/36	
Time			T0	T1	T0	T1
Mean chronological age	(Years), mean ± SD	Males + females	72.23 ± 3.82	73.28 ± 3.81	71.08 ± 4.10	72.10 ± 4.09
Mean BMI	kg/m^2^, mean ± SD		26.99 ± 3.60	26.67 ± 3.59	28.07 ± 3.37	28.02 ± 3.24
Adherence to NU-AGE diet	(According to NU-AGE diet score), mean ± SD		51.86 ± 9.78	64.84 ± 8.84	51.62 ± 9.52	66.69 ± 10.09
Mean chronological age	(Years), mean ± SD	Males	72.41 ± 3.91	73.48 ± 3.91	71.55 ± 4.27	72.58 ± 4.25
Mean BMI	kg/m^2^, mean ± SD		26.30 ± 2.88	25.79 ± 2.76	28.20 ± 3.06	28.19 ± 2.81
Adherence to NU-AGE diet	(According to NU-AGE diet score), mean ± SD		50.63 ± 10.43	66.48 ± 8.45	51.30 ± 8.39	66.74 ± 10.28
Mean chronological age	(Years), mean ± SD	Females	72.07 ± 3.80	73.12 ± 3.79	70.76 ± 4.01	71.78 ± 4.00
Mean BMI	kg/m^2^, mean ± SD		27.55 ± 4.05	27.39 ± 4.05	27.98 ± 3.61	27.91 ± 3.53
Adherence to NU-AGE diet	(According to NU-AGE diet score), mean ± SD		52.87 ± 9.25	63.49 ± 9.06	51.84 ± 10.31	66.66 ± 10.10

*BMI*, body mass index; *SD*, standard deviation

### Effect of the nutritional intervention on the epigenetic age acceleration measures

**Fig. 1:**
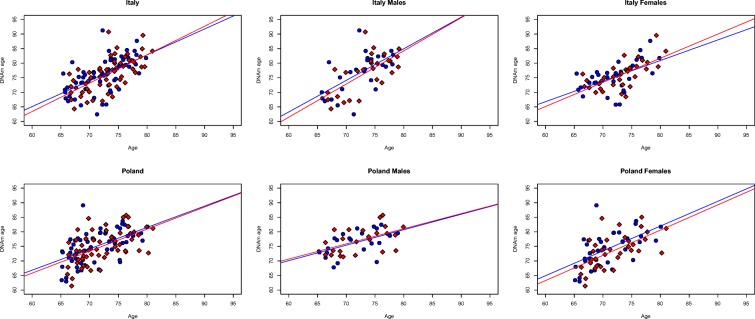
Significant association between DNAm age and chronological age at T0 and T1. Scatter plots of chronological age (*x*-axis) versus DNAm age (*y*-axis) in the different groups (T0 = blue; T1 = red). Lines represent fitted linear regressions

Epigenetic age (also referred to as DNA methylation age (DNAm age)) was calculated using the online age calculator freely available at the website: https://dnamage.genetics.ucla.edu. As expected, DNAm age was significantly associated with chronological age (*p* < 0.0001), both at T0 (before nutritional intervention) and at T1 (after a 12-month Mediterranean-like nutritional intervention), in both Italian and Polish groups (Fig. 1**)**.

For each subject, we evaluated the epigenetic age acceleration (AA), that is the deviation between DNAm age and effective chronological age. Positive values of epigenetic AA indicate an epigenetic age older than expected, while negative values indicate an epigenetic age younger than expected on the basis of chronological age. In particular, we considered three measures of epigenetic AA, implemented in the online age calculator, which reflect different aspects of epigenetic aging (see the “Subjects and methods” section): (1) AgeAccel; (2) Intrinsic Epigenetic Age Acceleration (IEAA); (3) Extrinsic Epigenetic Age Acceleration (EEAA) (Supplemental Table 1**)**. At T0, in Italian subjects, AgeAccel ranged from − 12.38 to 15.62 years, IEAA ranged from − 11.56 to 12.27 years, and EEAA ranged from − 10.90 to 7.41 years. In Polish subjects, AgeAccel ranged from − 8.49 to 16.27 years, IEAA ranged from − 9.01 to 15.55 years, and EEAA ranged from − 12.56 to 13.56 years. At T1, in Italian subjects, AgeAccel ranged from − 8.60 to 14.25 years, IEAA ranged from − 8.62 to 9.92 years, and EEAA ranged from − 8.97 to 7.90 years. In Polish subjects, at T1, AgeAccel ranged from − 9.81 to 10.99 years, IEAA ranged from − 10.44 to 9.47 years, and EEAA ranged from − 11.31 to 10.02 years. Baseline measures of epigenetic AA were similar between Italian and Polish subjects (Student’s *t* test *p* value > 0.05), but EEAA was significantly higher in Polish males compared with Polish females (Student’s *t* test *p* = 0.00009) and compared with Italian males (Student’s *t* test *p* = 0.02).

In both Italian and Polish cohorts, epigenetic AA measures at baseline were significantly associated (*p* < 0.05) with those obtained after the 12-month-tailored nutritional intervention (Supplemental Fig. 3).

**Fig. 2:**
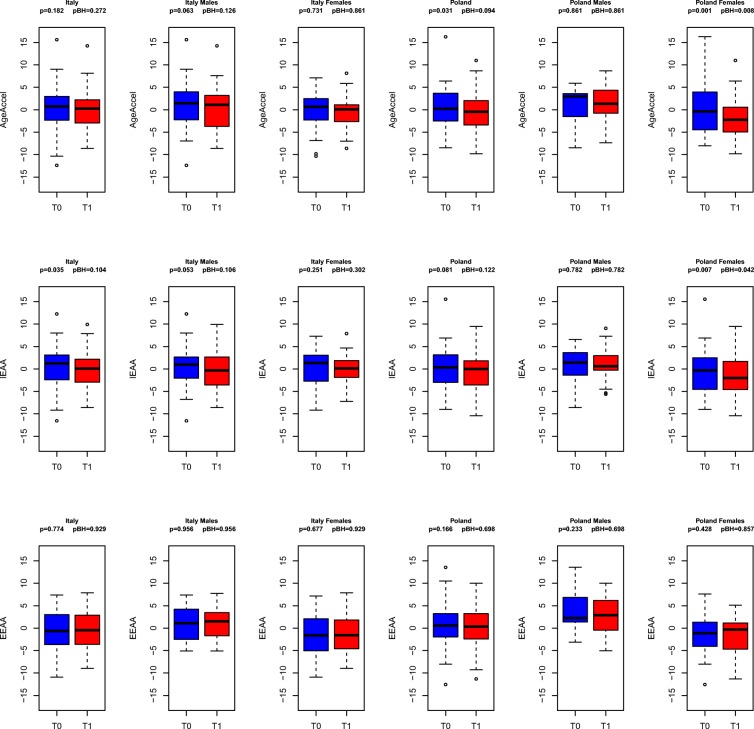
A 1 year Mediterranean-like diet intervention promotes epigenetic rejuvenation in a country- and sex-specific manner. Boxplots of epigenetic AA measures at T0 and T1 (upper panel, AgeAccel; middle panel, IEAA; lower panel, EEAA) considering all the subjects, only males, and only females. Uncorrected *p* values and *p* values after correction for multiple testing are displayed.

We then used Student’s paired-sample *t* test to compare the epigenetic AA measures at T0 and at T1. In Italian subjects, no statistically significant differences between T0 and T1 were observed considering AgeAccel, also when subjects were divided according to sex (Fig. 2, upper panel). On the contrary, in Polish subjects, AgeAccel measures were significantly lower at T1 versus baseline (T0) (*p* = 0.0312) (Fig. 2, upper panel). In other words, under the Mediterranean-like diet intervention, there was a statistically significant rejuvenation of the Polish subjects, according to the AgeAccel measure. When we divided samples on the basis of sex, we observed that the effect was predominantly related to a decrease in AgeAccel measures in Polish females at T1 compared with T0 (*p* = 0.0013). Rejuvenation of the Polish females after 1 year of nutritional intervention was confirmed with the IEAA measure (Fig. 2, middle panel), as analysis returned a significant decrease in IEAA values at T1 versus T0 (*p* = 0.007). Lower IEAA measures were also observed at T1 in Italian subjects as compared with T0 (*p* = 0.0347). The EEAA predictor did not give significant results (Fig. 2, lower panel) in both groups. After correction for multiple testing, the effect remained statistically significant for Polish females, according to AgeAccel (adjusted *p* value = 0.008) and IEAA (adjusted *p* value = 0.04) measures (Fig. 2).

Supplemental Fig. 4 reports, for each subject, the intra-pair difference between AgeAccel at T1 and AgeAccel at T0 (AgeAccel Diff), the intra-pair difference between IEAA at T1 and IEAA at T0 (IEAA Diff), and the intra-pair difference between EEAA at T1 and EEAA at T0 (IEAA Diff). In all three cases, a negative value indicates an epigenetic rejuvenation.

**Fig. 3:**
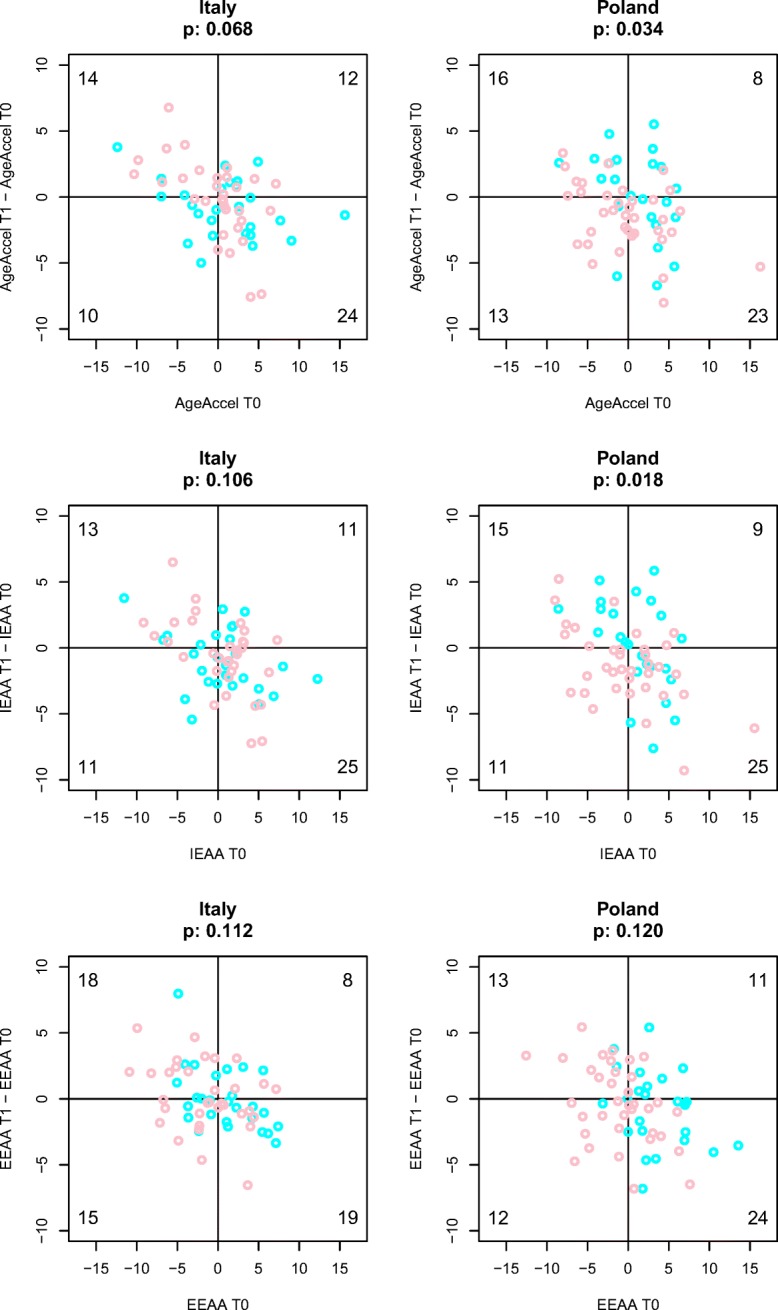
Stronger impact of diet on epigenetic AA measures in subjects with higher epigenetic AA values at baseline. Scatter plots of epigenetic AA measures at T1 (*x*-axis) versus epigenetic AA measures difference between T1-T0 (*y*-axis). The three epigenetic AA measures (AgeAccel, IEAA, and EEEA) are reported respectively in the upper, middle, and lower panels. Males and females subjects are indicated with cyan and pink circles, respectively. The number of subjects in each quadrant is reported. Fisher’s exact test was applied to test if there was a difference of proportion of subjects in the 4 quadrants

Finally, we assessed if AgeAccel Diff, IEAA Diff, and EEAA Diff values were related to, respectively, Age Accel, IEAA, and EEAA values at baseline (Fig. 3). In both the countries and for all the different AA measures, we found that the majority of subjects showing an epigenetic age rejuvenation (AgeAccel Diff, IEAA Diff, or EEAA Diff less than 0) had also baseline AA levels greater than 0 (Fig. 3). Fisher’s exact test confirmed that this enrichment was significant for AgeAccel and IEAA measures in Poles, indicating that the effect of the diet tended to be more marked in those subjects that displayed higher epigenetic AA values at T0.

### Association between epigenetic age acceleration measures, BMI, and adherence to the Mediterranean-like diet

In order to identify factors associated with the slowdown of the epigenetic AA measures, we first investigated the relationship between BMI and the epigenetic markers. We did not find any significant association between BMI and AgeAccel, IEEA or EEAA (results not shown).

We also analyzed the association between the epigenetic AA measures and the NU-AGE score measuring the adherence to the Mediterranean-like diet, calculated at T0 and T1 (see the “Subjects and methods” section). We observed a significant negative association of AgeAccel (*p* = 0.037) and IEAA (*p* = 0.027) with the NU-AGE score, with higher levels of adherence to the Mediterranean-like diet associated with negative epigenetic AA values, that is with epigenetic rejuvenation (Supplemental Fig. 5).

### Association between epigenetic age acceleration measures and genotype

**Fig. 4:**
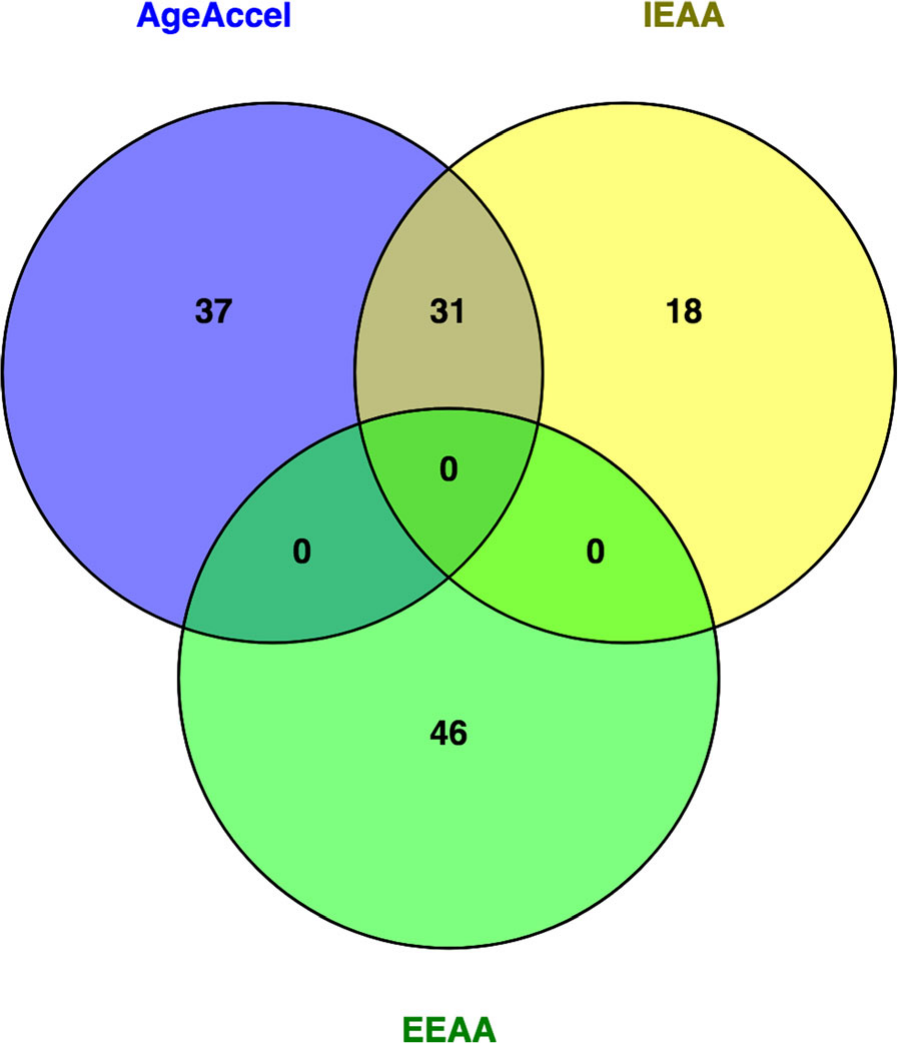
Thirty-one SNPs associated with AgeAccel are common with IEAA. Venn diagram of SNPs associated with AgeAccel (blue), IEAA (yellow), and EEAA (green)

Finally, we evaluated if a response to Mediterranean-like dietary intervention, in terms of epigenetic AA, was related to the genetic background of the participants of the study. To this aim, we carried out a genome-wide association study (GWAS) of epigenetic AA measures in our cohort, expressed as AgeAccel Diff, IEAA Diff, or EEAA Diff as described above (Supplemental Fig. 4). The quantile-quantile (QQ) plot of association results demonstrated no genomic inflation (data not shown). After correction for multiple testing, no significant association was observed at the genome-wide level (Benjamini-Hochberg corrected *p* value < 0.05). However, small-effect loci with nominal significance (*p* value < 1 × 10^−4^) were identified for all of the three measures of epigenetic AA. A total of 68, 49, and 46 single nucleotide polymorphisms (SNPs) were found significantly associated with AgeAccel Diff, IEAA Diff, and EEAA Diff, respectively (Supplemental Table 2). Thirty-one SNPs were common between AgeAccel Diff and IEAA Diff, while there were no SNPs in common between EEAA Diff and AgeAccel Diff or IEAA Diff (Fig. 4). Interestingly, 5 SNPs out of 68 (for AgeAccel Diff) and 6 SNPs out of 49 (for IEAA Diff) showed minor allele frequency differences between Italians and Polish (*p* values < 0.05).

In order to identify pathways that may be relevant to epigenetic AA effects upon Mediterranean-like nutritional intervention, we performed enrichment analysis using i-GSEA4GWAS (Supplemental Table 3). In the analysis of AgeAccel Diff associations, we found 60 significant gene sets (*p* < 0.05); 13 of which were significant after false discovery rate (FDR) correction (FDR < 0.05). IEAA Diff analysis returned 37 significant gene sets (*p* < 0.05); 11 of which had a FDR < 0.05. We found a large overlap between the enrichment analysis results of the two epigenetic AA measures, in particular for pathways involved in energy metabolism, regulation of cell cycle, and of immune functions. On the contrary, enrichment analysis for EEAA Diff did not return any significant result (*p* < 0.05).

## Discussion

In this study, we observed the effects of a 1-year Mediterranean-like diet, newly designed according to the nutritional needs of people over 65 years of age (Berendsen et al. 2014), on epigenetic AA measures. We analyzed blood methylation data, obtained in a population of subjects enrolled in a multicenter trial, and we demonstrated that the nutritional intervention delivered in the NU-AGE study can slow down the epigenetic aging rate of blood in specific groups of participants.

It is known that environmental factors, including diet, are able to modify the epigenome (Bacalini et al. 2014), and cross-sectional associations between epigenetic AA measures and diet have been previously described by Quach et al. (Quach et al. 2017). However, data from longitudinal studies on a possible rejuvenation of epigenetic age with dedicated therapeutic or lifestyle interventions are few. Only two works have been recently published on this topic. Firstly, Pavanello et al. examined the hypothesis that an intensive relaxing training of 60 days may influence epigenetic age by turning back the epigenetic clock (Pavanello et al. 2019) (Pavanello et al. 2019). They observed a trend to a reduction in DNAm age (estimated with the model proposed by Zbiec-Piekarska et al. (Zbieć-Piekarska et al. 2015) after training in six healthy subjects, but the effect was not statistically significant (*p* = 0.053). Secondly, the effect of a protocol intended to “rejuvenate the thymus” (thymus regeneration, immunorestoration, and insulin mitigation, TRIIM trial) was examined by Horvath’s team in a small, non-controlled study (Fahy et al. 2019). The 1-year intervention, composed of recombinant human growth hormone, dehydroepiandrosterone, and metformin, was delivered to 9 healthy aging men (age range 51–65 years old). A rejuvenating effect on four epigenetic age predictors (Horvath 2013; Hannum et al. 2013; Levine et al. 2018; Lu et al. 2019) was observed, with a mean change of about 2.5 years. The intervention was also associated with a protective effect on different immunosenescence biomarkers (reversal of thymic involution, increase in both naïve CD4+ and CD8+ T cells), and the effect persisted 6 months after discontinuing the treatment (Fahy et al. 2019).

Data regarding the impact of nutritional intervention are lacking, and to our knowledge, our study is the first longitudinal and interventional study to examine effects of such an intervention on epigenetic age acceleration measures in human blood cells. According to our results, a 1-year nutritional intervention could be able to rewind the epigenetic AA process in some specific groups. The discrepancy between the slowdown obtained with AgeAccel and IEAA measures on one hand, and the absence of effect observed with EEAA measure on the other hand seems to be of particular interest. Indeed, the three measures of epigenetic AA we studied in this work do not capture the same features of biological aging. By their very own construction, IEAA is considered as a measure of epigenetic age acceleration independent of age-related changes in the cellular composition of blood, whereas EEAA is more meant to capture the age-related decline of the immune system. Here, we did not observe any significant impact of the nutritional intervention on this decline according to the EEAA measure. Our results therefore suggest that the Mediterranean-like diet has a pure rejuvenating impact on the biological clock, and that this result is unconfounded by a potential effect of the intervention on the immune system.

Interestingly, the protective effect of the whole diet on the epigenetic age appears to be both country- and sex-specific, as Polish, and especially Polish females, appear to benefit the more for the intervention, according to the measures of epigenetic AA. Epigenetic aging rates have been previously described as influenced by race/ethnicity (Horvath et al. 2016) and sex (Horvath et al. 2016; Xiao et al. 2018), and we also demonstrated here that the epigenetic response to an intervention can be influenced by these parameters. It is likely that the observed differences between males and females are related not only to pure biological differences (for example, differences in body composition (Santoro et al. 2018), but also to anthropologic and cultural components (such as levels of education, cooking, or willingness to stick to the nutritional advices for example).

While population and sex-specificities appear clearly in this work, inter-individual differences intervene also in the response to the nutritional intervention. Firstly, subjects that were epigenetically older at baseline (i.e., subjects with higher epigenetic AA values at T0) had a more marked effect of the nutritional intervention and seemed to benefit more of the effects of the Mediterranean-like diet. Secondly, according to our GWAS, some genetic variants influence the response to the intervention. GWAS results were largely overlapping between AgeAccel and IEAA analysis. Furthermore, enrichment analysis suggested that both epigenetic AA measures were associated to genetic variants in genes involved in pathways related to the regulation of cell metabolism and immune function. Among the gene sets associated to AgeAccel differences between T1 and T0, it is worth to note the presence of the mTOR pathway, which plays a pivotal role in the regulation of nutrients-sensing and energy metabolism during aging (Cummings and Lamming 2017; Tosti et al. 2018; Lushchak et al. 2019). In animal models, it has been previously demonstrated the influence of a genetic component in the response to a nutritional intervention, such as caloric restriction (CR) (Liao et al. 2010). Liao et al. observed that lifespan expansion by CR was not universal in mice and was highly dependent on the strain of the animals, suggesting the important influence of the genotype in the CR effect (Liao et al. 2010). In humans, this problem has been poorly investigated. Previous reports have evaluated the association between genetic background and epigenetic AA in different tissues (Lu et al. 2016, 2017, 2018), but little is known about the influence of genetics on the response to a nutritional intervention. In a recent study, the effects of dietary supplementation with folic acid and vitamin B12 on epigenetic age deceleration were found dependent upon gender and *MTHFR* genotype (Sae-Lee et al. 2018). Only the group of women with the *MTHFR* 677CC genotype displayed a deceleration in epigenetic aging upon vitaminic supplementation (Sae-Lee et al. 2018).

In summary, strengths of our study include the following: (1) the experimental design (multi-center trial with 1-year follow-up), with the inclusion of two different populations coming from one Mediterranean and one non-Mediterranean European countries, with extensive assessment at baseline and after one-year of intervention; (2) a well-controlled nutritional intervention; (3) the combination of data on genetics and epigenetics for the same individuals; and (4) the use of the state-of-the-art epigenetic biomarkers of aging, which are robust and well-validated instruments, previously associated with mortality, morbidity, and several age-related phenotypes (Horvath and Raj 2018).

On the contrary, several important limitations exist. Firstly, the absence of a control group is a major one. Genome-wide DNA methylation analysis was performed only in a subset of subjects included in the intervention group and was not performed on subjects randomized into the control group. This outline corresponds to the initial design of the NU-AGE study, as described by Santoro et al. (Santoro et al. 2014), where it was originally decided to perform OMICs analysis (epigenomics, transcriptomics, metabolomics, and metagenomics) in a subgroup of 120 randomly selected subjects before and after diet. There were no specific changes in the lifestyle behaviors of the individuals recruited in the control group, and specially, no significant increase in the NU-AGE index, which measures the adherence to Mediterranean diet. This index, while not different between the two groups at baseline, only changed significantly in the intervention group after 1 year, as compared with the control one (Berendsen et al. 2018). In support of our data, it has been demonstrated that Mediterranean diet can have a specific impact on DNA methylation levels of certain genes. Thus, in the PREDIMED study, it was observed that a nutritional intervention based on Mediterranean diet was able to impact on DNA methylation levels of inflammation-related genes compared with the non-intervention group (Arpón et al. 2017).

Secondly, in our experimental settings, the nutritional intervention resulted only in a small effect size (Supplementary Table 1), which however is reasonable according to recent literature. In the recently published results of the TRIIM trial (Fahy et al. 2019), the nine volunteers were found to be on average 2.5 years younger after the intervention than they entered, which means that they gained back about 1.5 years in 1 year of trial. It is reasonable that the effect of NU-AGE nutritional intervention (i.e., average of − 1.47 years of AgeAccelDiff and of − 1.36 years of IEAADiff in the subgroup of Polish females) could have a minor effect on aging biomarkers compared with the one presented by Fahy et al., who evaluated the impact of 3 combined drugs. However, this small effect should be necessarily confirmed in a larger number of enrolled subjects compared with the cohort analyzed in our pilot study. The same applies to our GWAS analysis, as no significant association was observed after correction for multiple testing and as the small sample size prevented us from performing the analysis in subgroups divided for country or sex.

In conclusion, we report that a Mediterranean-like nutritional intervention can promote epigenetic rejuvenation in the elderly, and that its effect is dependent on different factors including the following: (1) country-/population-specific factors, likely influenced by anthropologic and cultural components; (2) sex-/gender-specific factors: and (3) individual-specific factors, for example, related to the genetic background and to the baseline epigenetic profile of each individual.

Further work is required to overcome the important limitations of this preliminary work, to elucidate how some specific determinants influence the epigenetic aging and how some individuals seem to be more prone to benefit from specific interventions. This will be a key achievement for the development of individualized nutritional interventions aimed at promoting healthy living and, more in general, for the application of a precision medicine approach to anti-aging interventions.

## Electronic supplementary material

ESM 1 (PDF 6 kb)

ESM 2 (PDF 27 kb)

ESM 3 (PDF 8 kb)

ESM 4 (PDF 7 kb)

ESM 5 (PDF 10 kb)

ESM 6 (DOCX 17 kb)

ESM 7 (XLSX 28 kb)

ESM 8 (XLSX 14 kb)
