# Supplementary figures and images for: One-year Mediterranean diet promotes epigenetic rejuvenation with country- and sex-specific effects: a pilot study from the NU-AGE project
Source: GeroScience. 2020 Jan 24;42(2):687–701. doi: 10.1007/s11357-019-00149-0 (PMC7205853; doi:10.1007/s11357-019-00149-0)

Italy

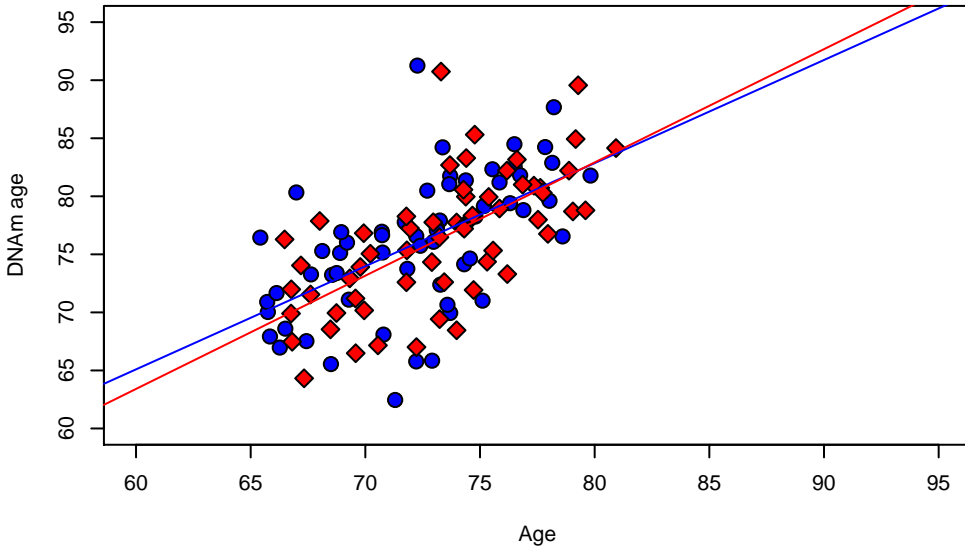

Italy Males

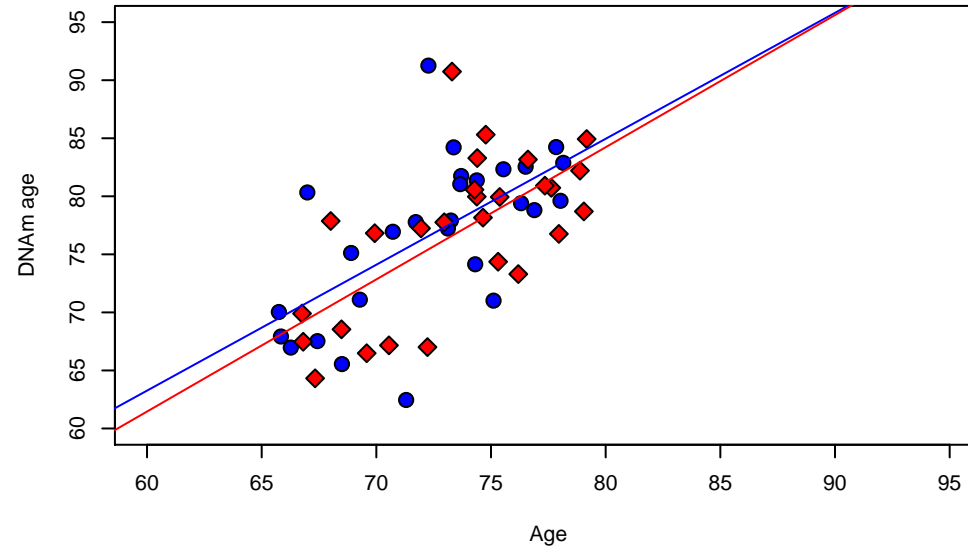

Italy Females

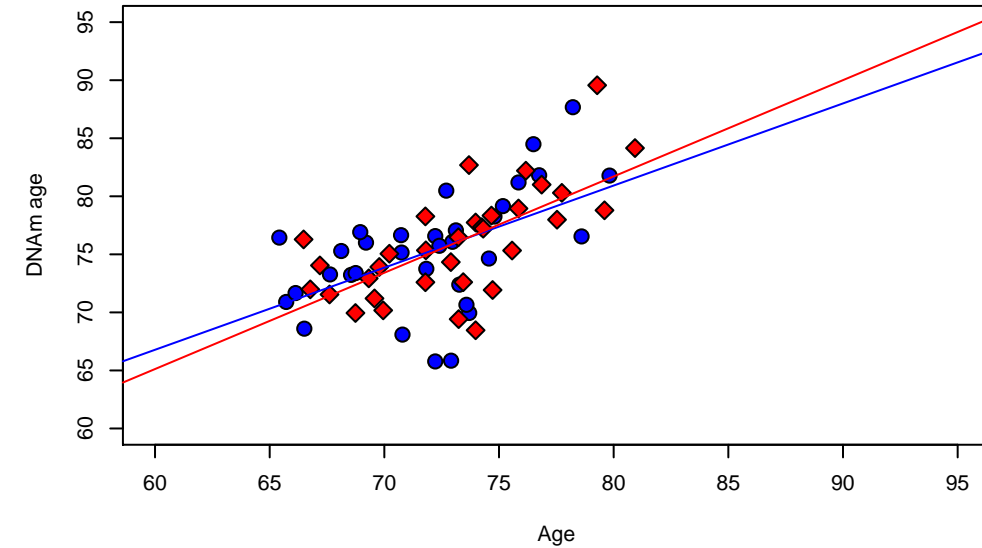

Poland

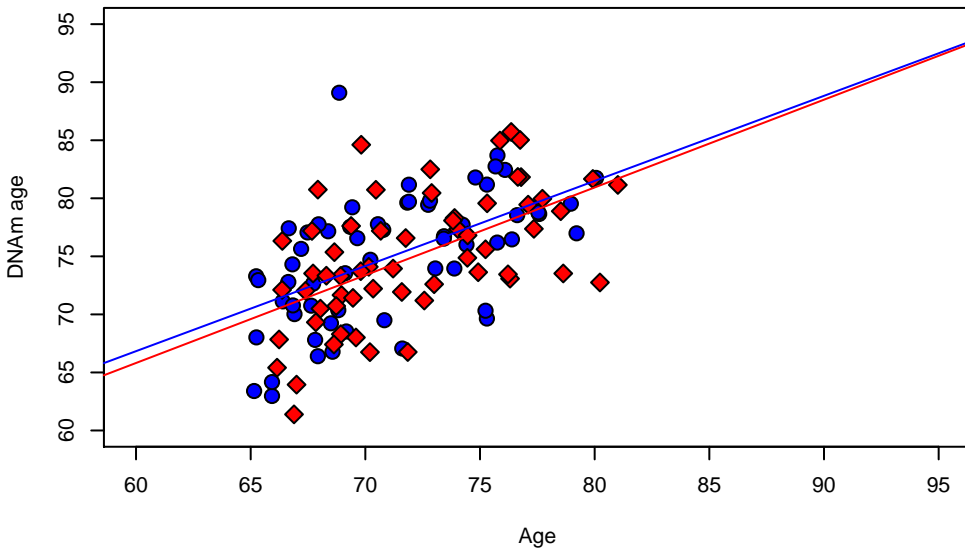

Poland Males

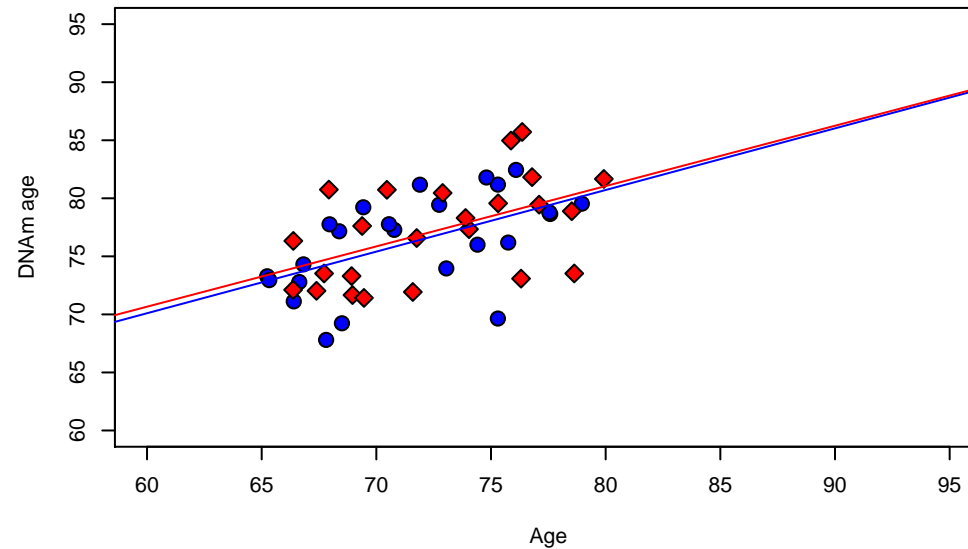

Poland Females

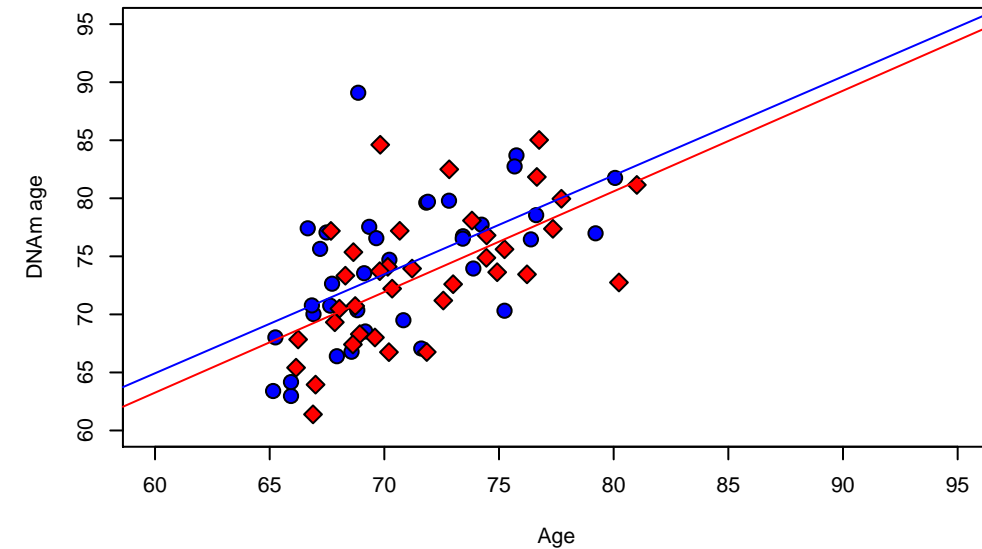

Supplement: Supplementary file 1 — (PDF 6 kb) [file 11357_2019_149_MOESM1_ESM.pdf]

**Males + Females**

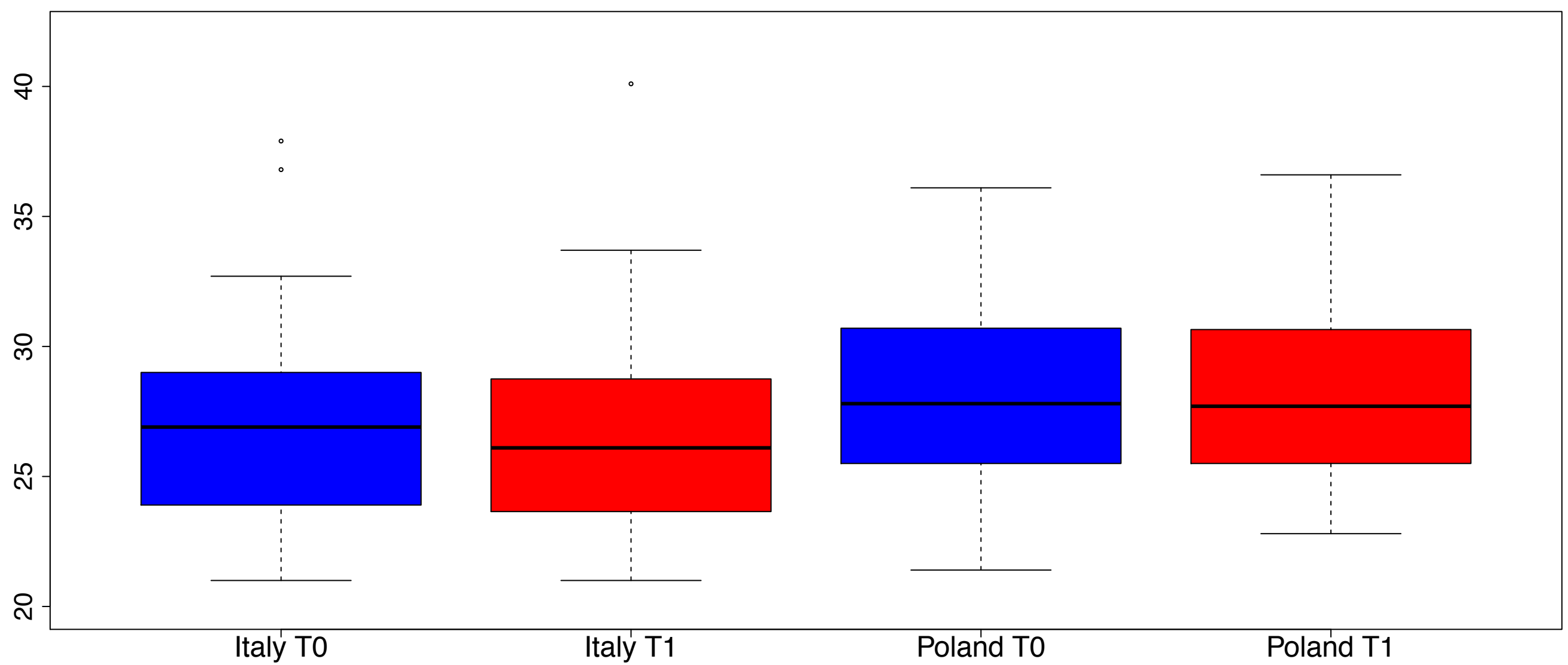

**Males**

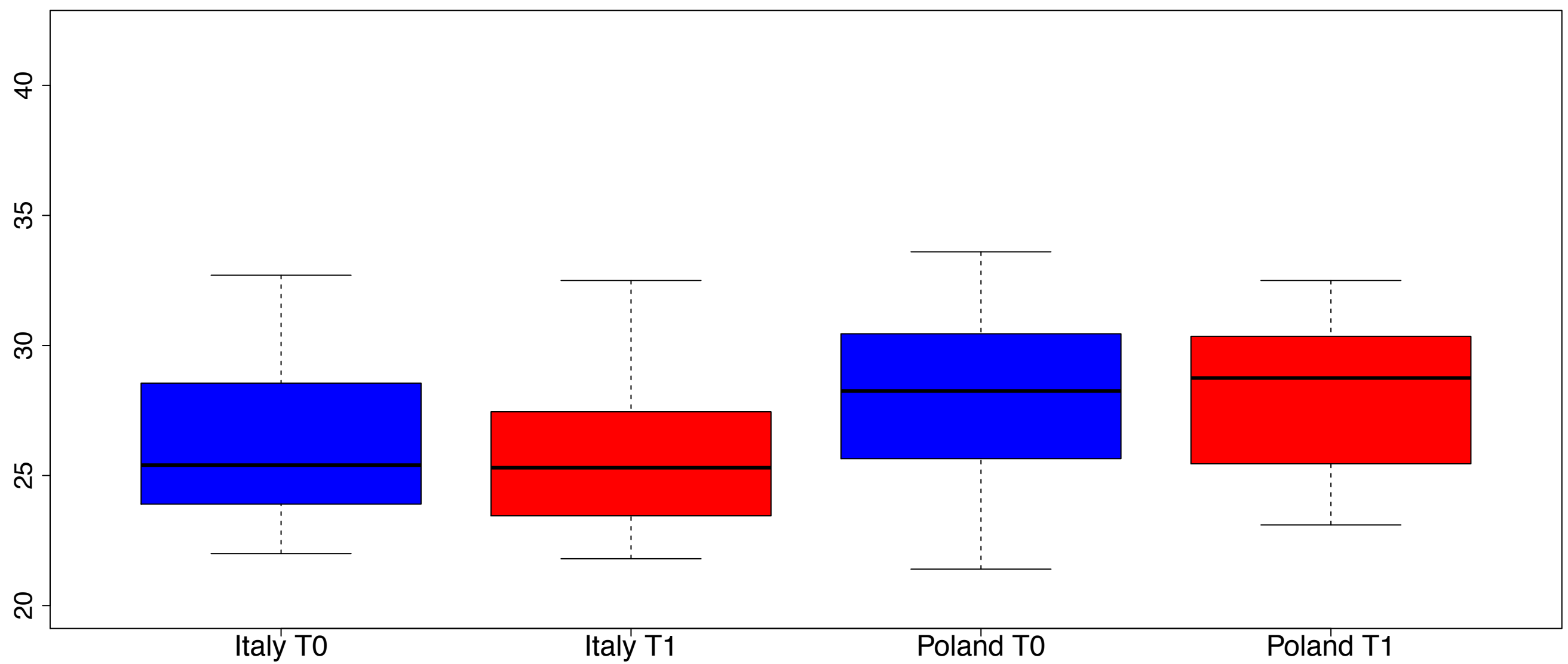

**Females**

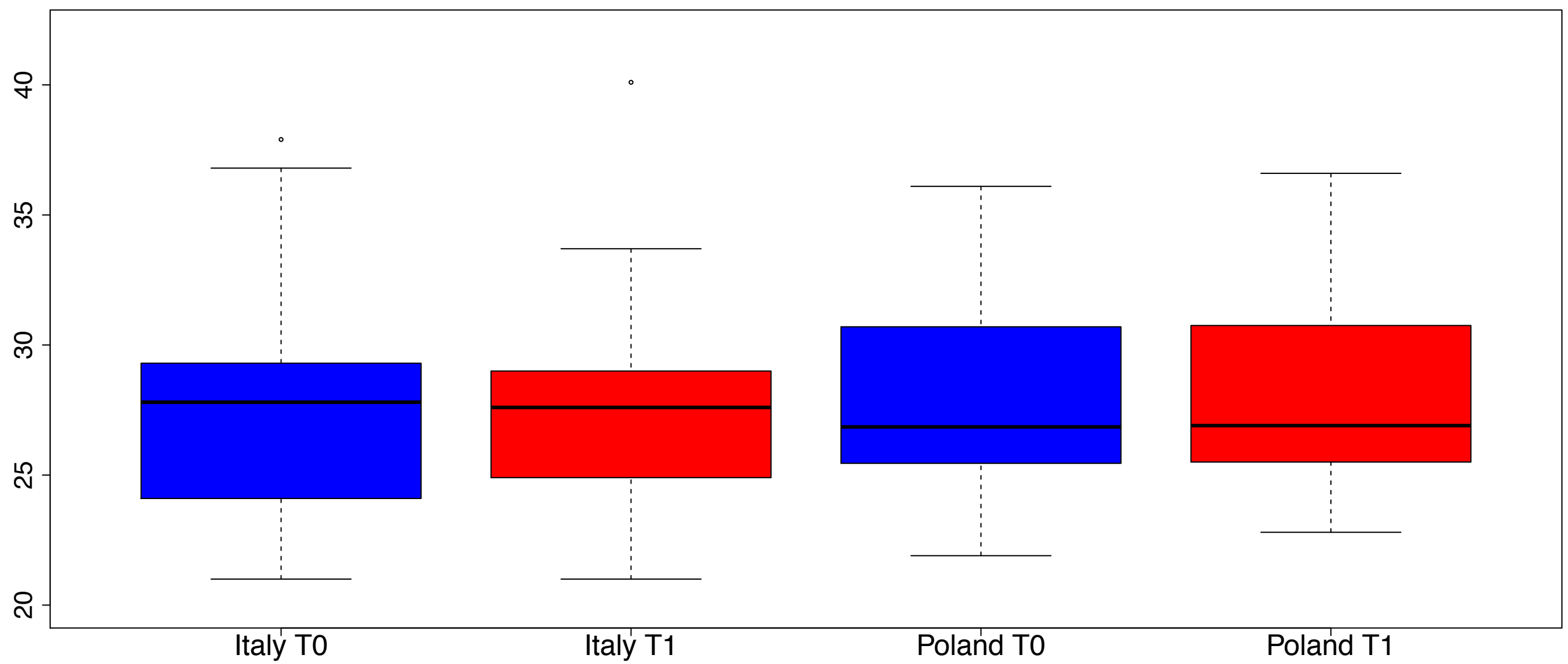

Supplement: Supplementary file 2 — (PDF 27 kb) [file 11357_2019_149_MOESM2_ESM.pdf]

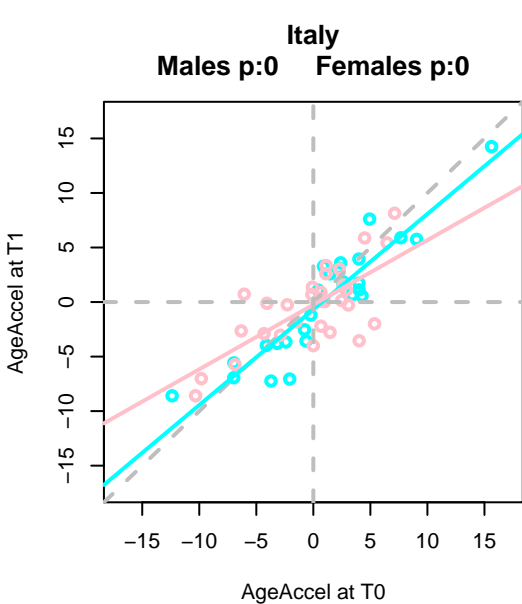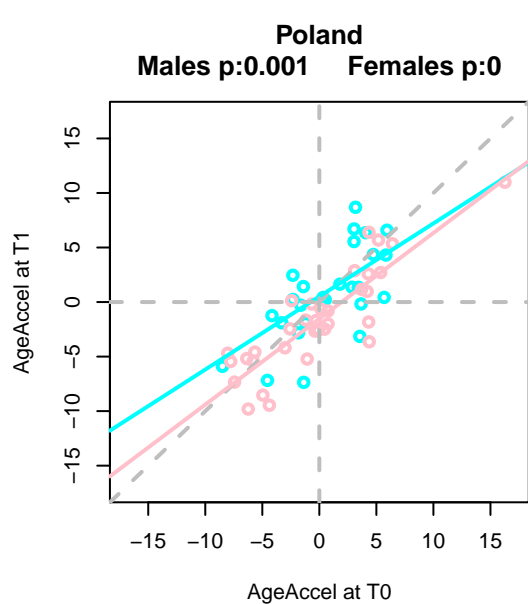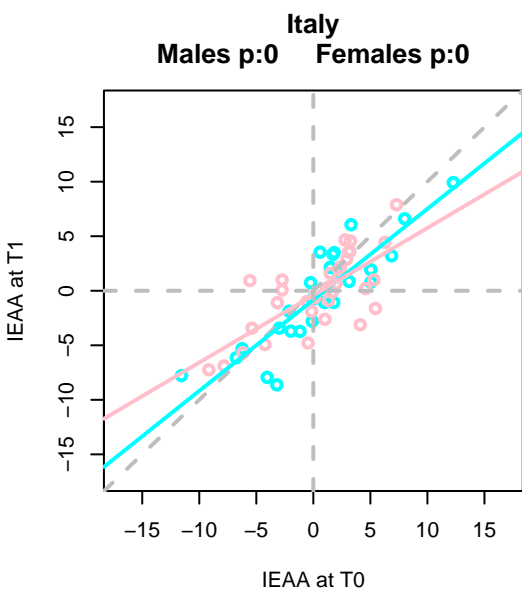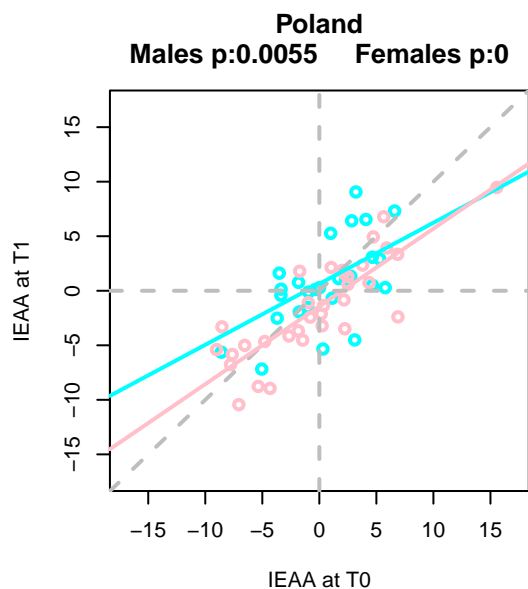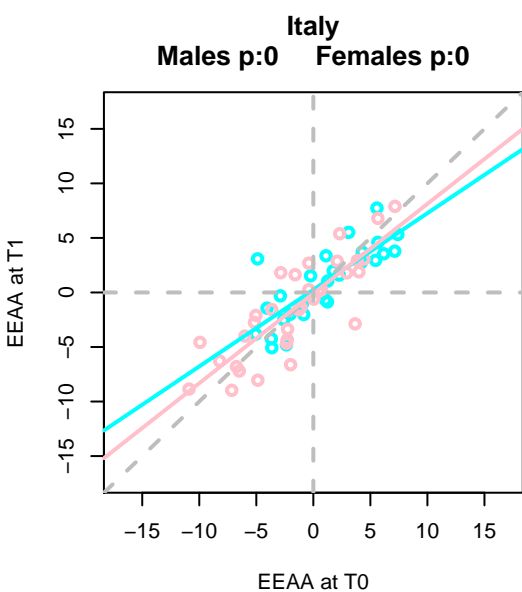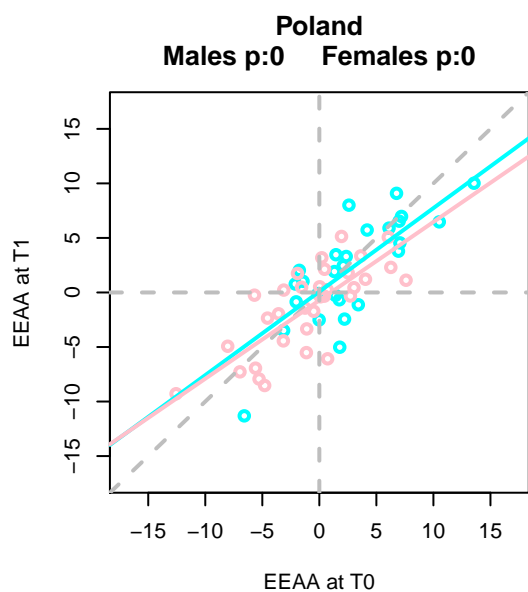

Supplement: Supplementary file 3 — (PDF 8 kb) [file 11357_2019_149_MOESM3_ESM.pdf]

Italy AgeAccel

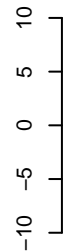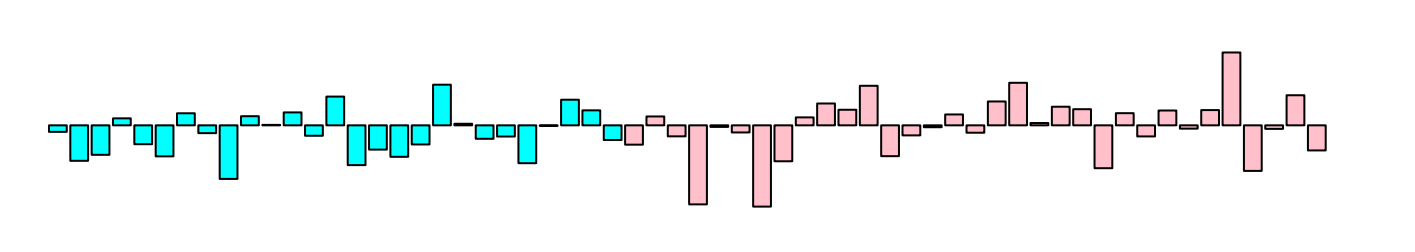

Poland AgeAccel

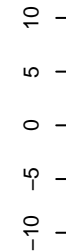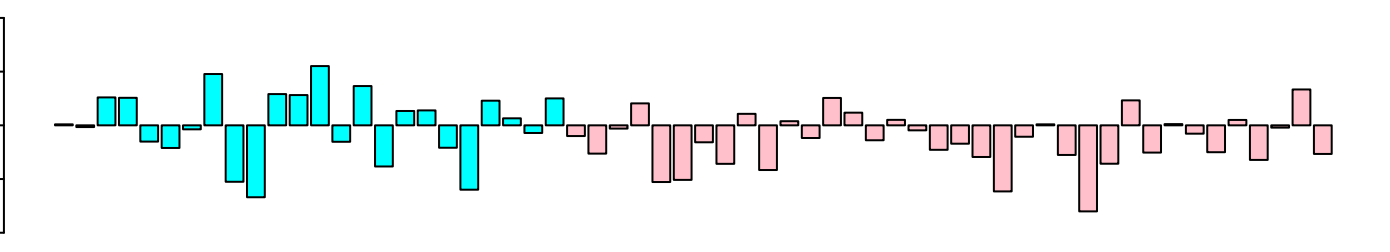

Italy IEAA

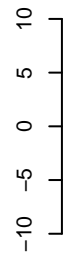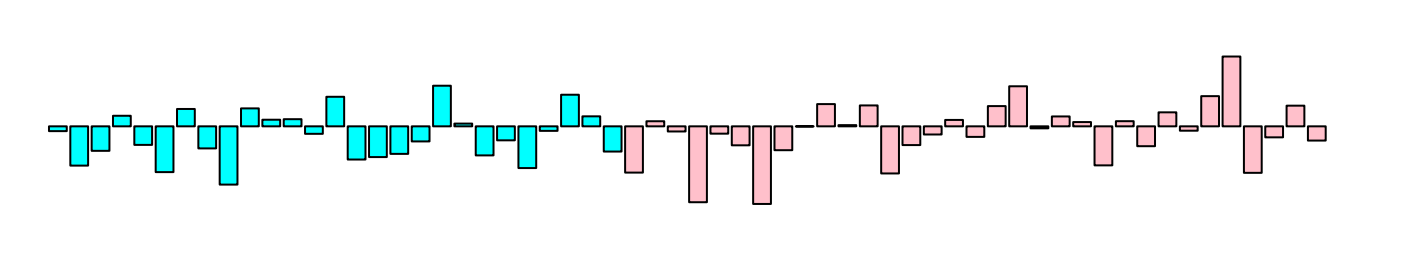

Poland IEAA

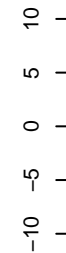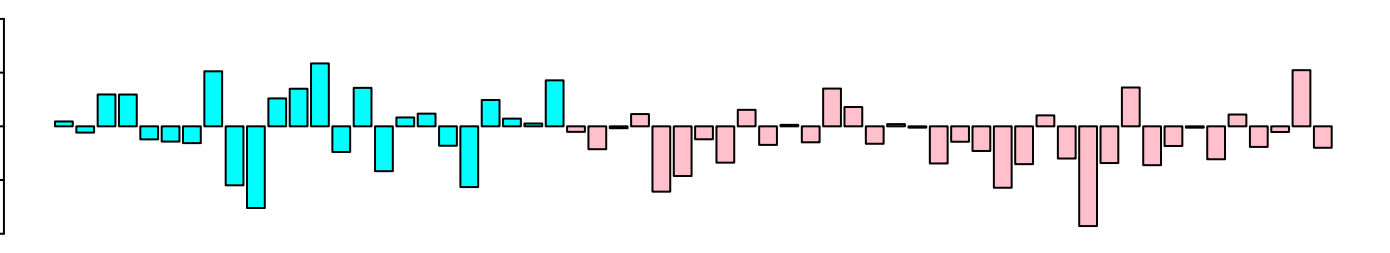

Italy EEAA

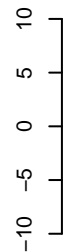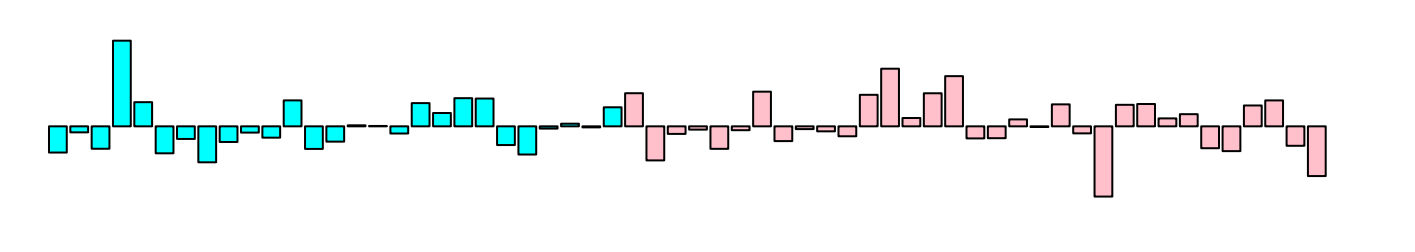

Poland EEAA

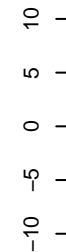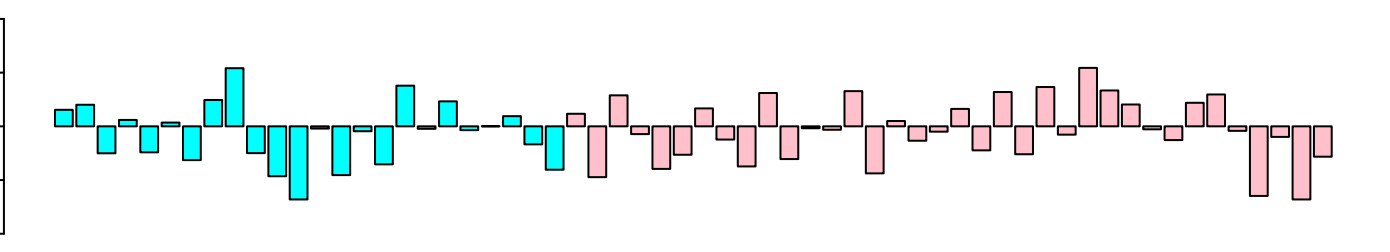

Supplement: Supplementary file 4 — (PDF 7 kb) [file 11357_2019_149_MOESM4_ESM.pdf]

AgeAccel

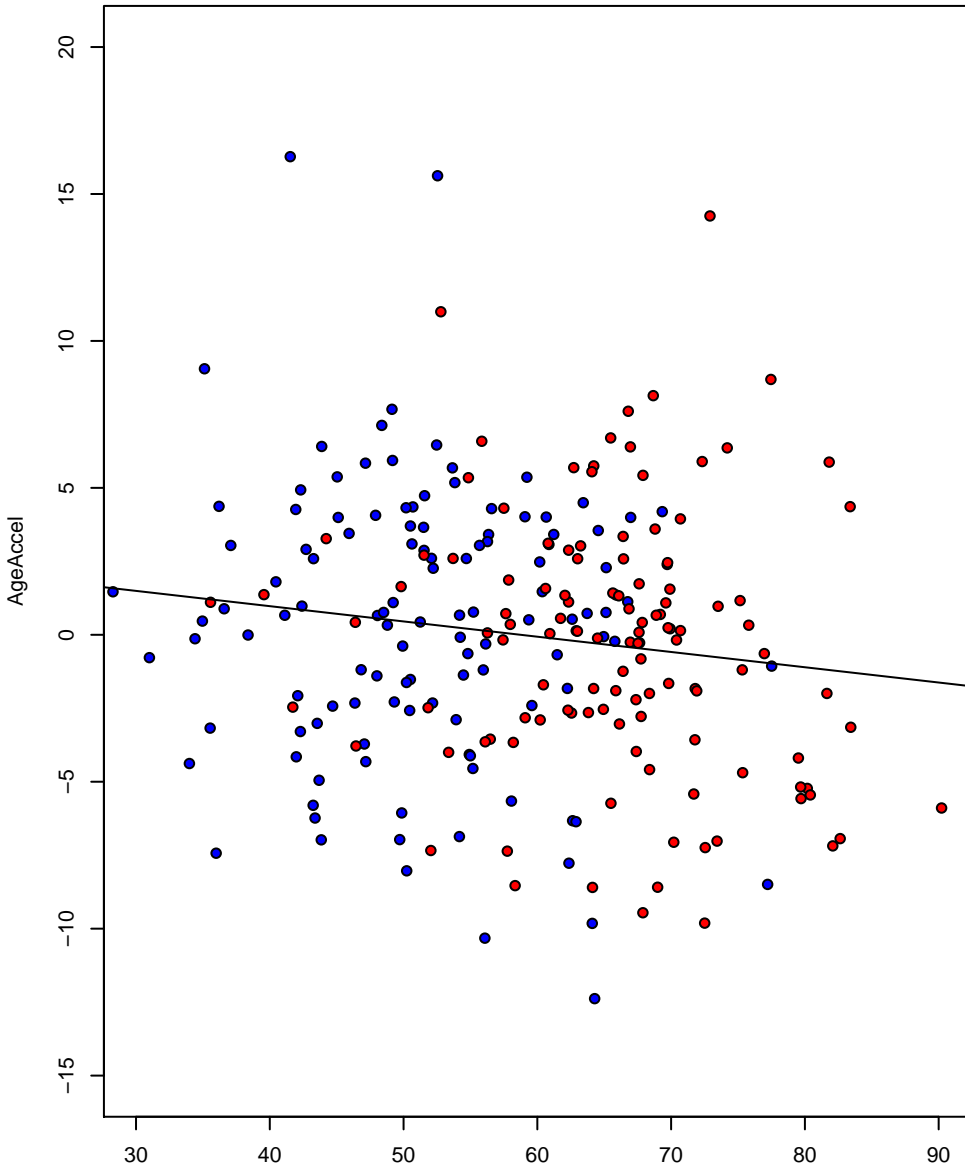

NU-AGE score  
pvalue: 0.0372021130806068

IEAA

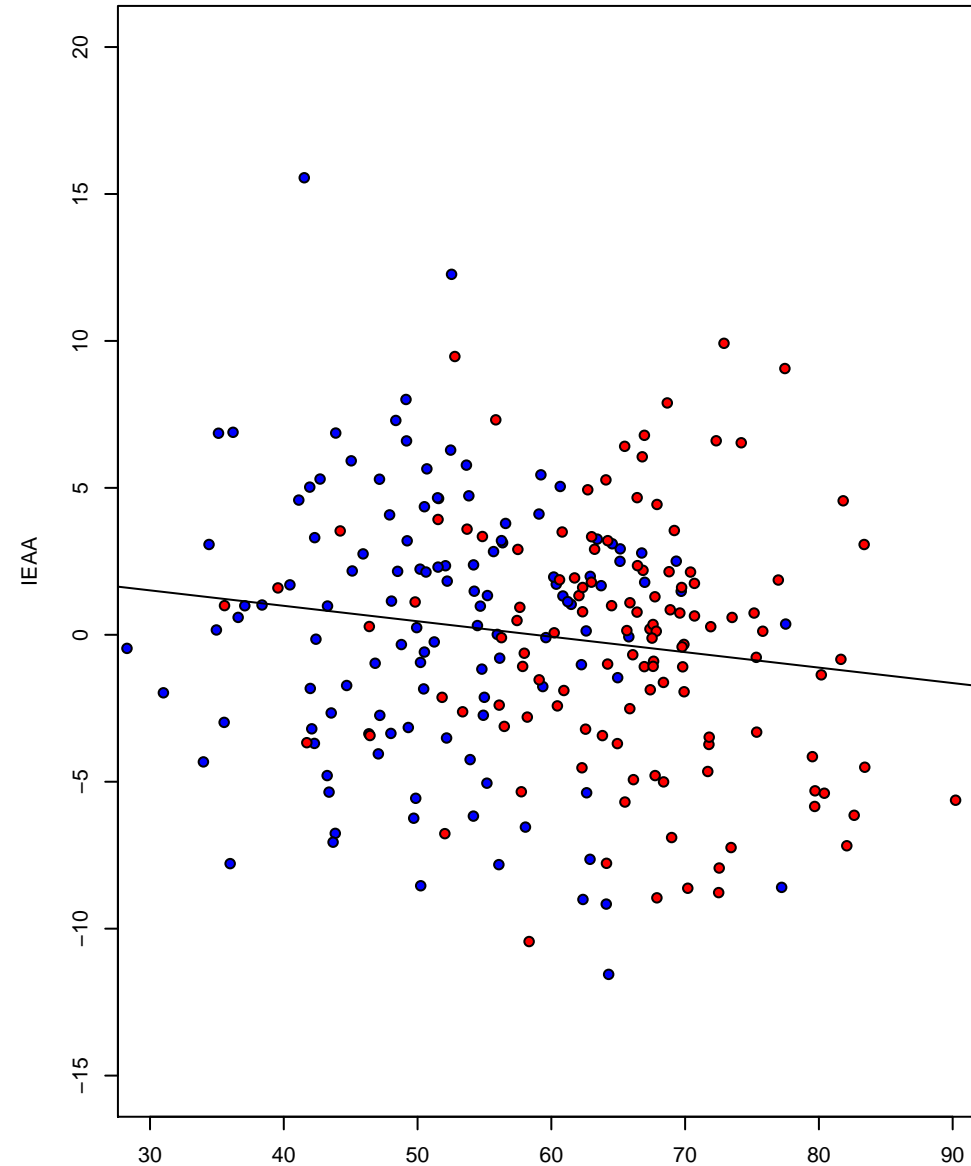

NU-AGE score  
pvalue: 0.0274886909111795

EEAA

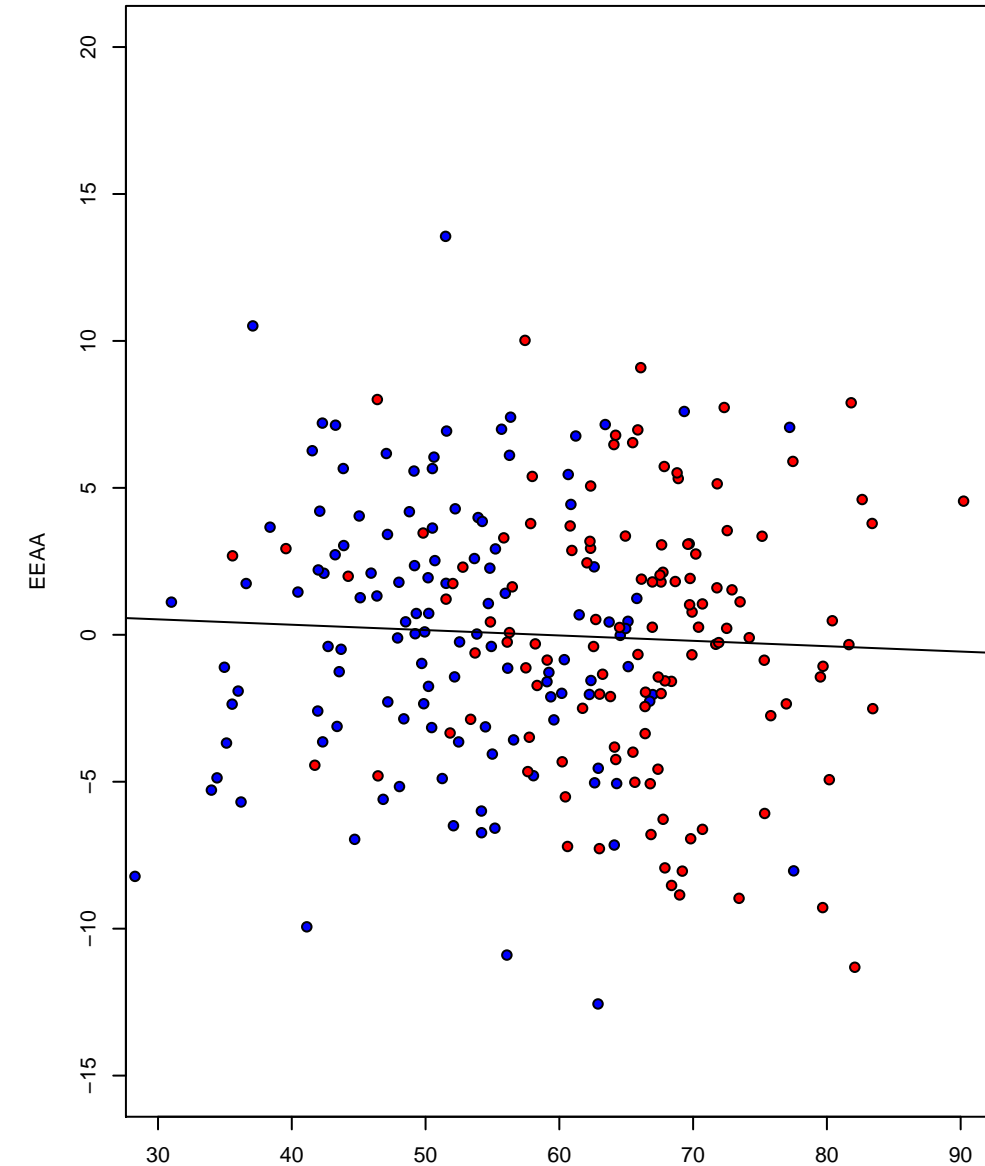

NU-AGE score  
pvalue: 0.456490131563061

Supplement: Supplementary file 5 — (PDF 10 kb) [file 11357_2019_149_MOESM5_ESM.pdf]
