# Supplementary material for: One-year Mediterranean diet promotes epigenetic rejuvenation with country- and sex-specific effects: a pilot study from the NU-AGE project
Source: GeroScience. 2020 Jan 24;42(2):687–701. doi: 10.1007/s11357-019-00149-0 (PMC7205853; doi:10.1007/s11357-019-00149-0)
Supplement: Supplementary file 6 — (DOCX 17 kb) [file 11357_2019_149_MOESM6_ESM.docx]

| **Country** | |  | **Italy** | | **Poland** | |
| --- | --- | --- | --- | --- | --- | --- |
| **Subjects (n)** | |  | 60 | | 60 | |
| **Males / Females (n)** | |  | 27 / 33 | | 24 / 36 | |
| **Time** | |  | **T0** | **T1** | **T0** | **T1** |
| **DNAm age** | (years), mean ± SD | **Males + Females** | 75.95 ± 5.9 | 76.36 ± 5.7 | 74.95 ± 5.5 | 74.96 ± 5.5 |
| **AgeAccel** | (years), mean ± SD |  | 0.35 ± 4.9 | -0.12 ± 4.3 | 0.30 ± 4.6 | -0.54 ± 4.6 |
| **IEAA** | (years), mean ± SD |  | 0.49 ± 4.4 | -0.24 ± 4.0 | 0.24 ± 4.7 | -0.49 ± 4.4 |
| **EEAA** | (years), mean ± SD |  | -0.48 ± 4.4 | -0.39 ± 4.2 | 0.70 ± 4.7 | 0.16 ± 4.6 |
| **AgeAccelDiff** | (years), mean ± SD |  | -0.46 ± 2.6 | | **-0.84 ± 2.9** | |
| **IEAADiff** | (years), mean ± SD |  | **-0.74 ± 2.6** | | -0.74 ± 3.2 | |
| **EEAADiff** | (years), mean ± SD |  | 0.09 ± 2.5 | | -0.53 ± 3.0 | |
| **DNAm age** | (years), mean ± SD | **Males** | 76.72 ± 6.9 | 76.80 ± 6.8 | 76.23 ± 4.2 | 77.20 ± 4.3 |
| **AgeAccel** | (years), mean ± SD |  | 0.97 ± 5.5 | 0.17 ± 5.2 | 1.18 ± 3.7 | 1.30 ± 3.9 |
| **IEAA** | (years), mean ± SD |  | 0.55 ± 4.8 | -0.38 ± 4.6 | 0.99 ± 3.7 | 1.19 ± 3.8 |
| **EEAA** | (years), mean ± SD |  | 0.90 ± 3.9 | 0.88 ± 3.4 | 3.47 ± 4.0 | 2.73 ± 4.1 |
| **AgeAccelDiff** | (years), mean ± SD |  | -0.80 ± 2.1 | | 0.12 ± 3.3 | |
| **IEAADiff** | (years), mean ± SD |  | -0.92 ± 2.4 | | 0.20 ± 3.5 | |
| **EEAADiff** | (years), mean ± SD |  | -0.03 ± 2.4 | | -0.74 ± 2.9 | |
| **DNAm age** | (years), mean ± SD | **Females** | 75.32 ± 5.0 | 76.00 ± 4.7 | 74.10 ± 6.1 | 73.47 ± 5.7 |
| **AgeAccel** | (years), mean ± SD |  | -0.16 ± 4.3 | -0.34 ± 3.5 | -0.29 ± 5.1 | -1.76 ± 4.6 |
| **IEAA** | (years), mean ± SD |  | 0.45 ± 4.1 | -0.13 ± 3.5 | -0.26 ± 5.3 | -1.61 ± 4.5 |
| **EEAA** | (years), mean ± SD |  | -1.6 ± 4.5 | -1.4 ± 4.5 | -1.14 ± 4.3 | -1.54 ± 4.1 |
| **AgeAccelDiff** | (years), mean ± SD |  | -0.18 ± 3.0 | | ***-1.47 ± 2.5*** | |
| **IEAADiff** | (years), mean ± SD |  | -0.58 ± 2.9 | | ***-1.36 ± 2.9*** | |
| **EEAADiff** | (years), mean ± SD |  | 0.19 ± 2.6 | | -0.40 ± 3.0 | |

**Supplementary Table 1**: **DNAm age and measures of epigenetic age acceleration at baseline (T0) and after one year of Mediterranean-like diet (T1).**

SD: standard deviation. DNAm age: DNA methylation age. IEAA: Intrinsic Epigenetic Age Acceleration. EEAA: Extrinsic Epigenetic Age Acceleration. AgeAccelDiff: For each individual, AgeAcceleration difference between T1 and T0. IEAADiff: For each individual, IEAA difference between T1 and T0. EEAADiff: For each individual, EEAA difference between T1 and T0.

Statistically significant differences in epigenetic AA measures between T0 and T1 are in bold (nominal p-value) and in italic (BH-adjusted p-value).
